# Cell-cycle and suppressor proteins expression in uterine cervix in HIV/HPV co-infection: comparative study by tissue micro-array (TMA)

**DOI:** 10.1186/1471-2407-8-289

**Published:** 2008-10-07

**Authors:** Alcina F Nicol, Andréa Rodrigues Cordovil Pires, Simone R de Souza, Gerard J Nuovo, Beatriz Grinsztejn, Aparecida Tristão, Fabio B Russomano, Luciane Velasque, José R Lapa e Silva, Claude Pirmez

**Affiliations:** 1 Laboratory of Immunopathology, Instituto Oswaldo Cruz – FIOCRUZ, Rio de Janeiro, Brazil; 2 Fonte Medicina Diagnostica Laboratory, Niterói, and Federal Fluminense University, Niterói, Rio de Janeiro, Brazil; 3 Department of Pathology, University Hospitals, Columbus, Ohio, USA; 4 Department of Infectious Disease of Evandro Chagas Clinical Research Institute, FIOCRUZ, Rio de Janeiro, Brazil; 5 Department of Mathematics and Statistics of Federal University of Rio de Janeiro, Rio de Janeiro, Brazil; 6 Fernandes Figueira Institute, FIOCRUZ, Rio de Janeiro, Brazil; 7 Multidisciplinary Laboratory, Clementino Fraga Filho University Hospital, Federal University of Rio de Janeiro, Rio de Janeiro, Brazil

## Abstract

**Background:**

The oncoproteins of human papillomavirus (HPVs) directly effect cell-cycle control. We hypothesize that regulatory and cell cycle protein expression might be additionally modified in the cervix of HIV/HPV co-infected women.

**Methods:**

We analyzed the expression of Rb, p27, VEGF and Elf-1 transcriptor factor by immunohistochemistry in 163 paraffin-embeded cervical samples using Tissue Micro-Array (TMA) and correlated this to HIV-1 and HPV infection.

**Results:**

HIV/HPV co-infection was associated with a significant increase in expression (p < 0.001) of VEGF and p27 in both low and high grade CIN when compared to the cervices of women infected by HPV alone. Decreased Rb expression was evident with increased CIN grade in the cervices of women infected with HPV alone (p = 0.003 average of cells/mm^2 ^in CIN I: 17.9, CIN II/III: 4.8, and tumor 3.9). Rb expression increased 3-fold for both low and high grade CIN with HPV/HIV-1 co-infection compared to HPV infection alone but did not reach statistical significance. There was a significant increase in Elf-1 expression in HPV+/HIV- women with CIN II/III and tumor (average of cells/mm^2 ^in CIN I: 63.8; CIN II/III: 115.7 and tumor: 112.0, p = 0.005), in comparison to controls.

**Conclusion:**

Co-infection of HPV and HIV leads to significant increase in the VEGF and p27 expression when compared to HPV+/HIV-negative infection that could facilitate viral persistence and invasive tumor development.

## Background

Human papillomavirus (HPVs) are DNA viruses that produce hyperproliferative lesions in epithelial tissues. High-risk HPV genital infection, in particular HPV types 16 and 18, is associated with the development of genital cancer, while the low-risk HPVs induce benign genital warts. The oncogenic potential of HPV is, in part, mediated by the E6 and E7 proteins, which are known to bind and inactivate the p53 and retinoblastoma protein (pRb) respectively. Rb is one of the most investigated tumor suppressor genes and its dysregulation has been associated with neoplastic growth in a variety of tumors [1]. The ability of HPV oncoproteins to disrupt growth regulatory proteins may have effects on the cyclin-dependent kinase (CDK) inhibitors linked to the G1- and G2- checkpoints. A group of CDK inhibitors, which includes p21, p27 and p57, inhibits the cyclin/cdk2, cdk4 complexes and causes G1 arrest. These proteins are rarely mutated in human tumors and their expression levels is of prognostic significance in a wide spectrum of tumors [2]. P27 may be inactivated upon binding to DNA tumor virus oncoprotein, such as HPV 16 E7, can be deregulated early in carcinogenesis and may precede tumor invasion. Other HPV proteins act indirectly by altering the function of cellular factors. The loss of cell-cycle control leads to increased proliferation that can be detected by the increased number of cells in the cycle expressing specific markers [3].

Among the proteins and factors involved in cellular growth, VEGF (vascular endothelial growth factor) is a potent angiogenic factor that operates as an angiogenesis tumor mediator [4]. The role of VEGF in cervical carcinogenesis has been studied and it is established that the VEGF expression has been related to microvascular density (MVD) in cervical intraepithelial neoplasia (CIN) and invasive carcinomas, suggesting that VEGF is an early marker of cervical carcinogenesis, with linearly increasing expression starting from low grade CIN [4]. Another factor is eukaryotic translation initiation factors (Ets), which includes Elf, a lymphoid-specific member of this family, known to regulate inducible gene expression during T cell activation which includes increased angiogenesis. An over-expression of these factors can lead to neoplastic cellular change and tumor. Recent report supported a role of Elf-1 in the development of tumor angiogenesis and showed that targeting of Elf-1 in melanoma tumors resulted in decreased angiogenesis [5].

There is still relatively little data on biomarkers that may relate to increased risk of progression of CIN to cervical cancer [6,7] however, there is evidence that a few markers can be used as more specific risk assessment tools [8].

HIV/HPV co-infection is associated with a marked increase risk in progression of CIN to cervical cancer [9]. Although the reasons for this observation are not well understood, cytokine profile alterations, including RANTES, have been described in co-infected patients [10].

Data suggests that HPV infection of the cervix might influence HIV pathogenesis by inducing both immune and inflammatory related protein production that enhances HIV expression [11].

Recent insights into molecular pathogenesis have demonstrated that HIV-1 proteins can directly lead to cancer growth by interfering with cellular functions. Also, HIV-1 proteins such as Tat can interact with the RB2/p130 tumor suppressor gene product and induce increased cell proliferation [12]. However the mechanisms whereby HIV-1 subverts cell-cycle controls in the infected cervix have been unclear. Despite the fact that some reports have shown a role of VEGF, Rb, p27 in the HPV cervical CIN progression, little is known about the involvement and expression of these proteins in the HIV/HPV co-infection.

We investigated the hypothesis that HIV would modify the expression of regulatory and cell-cycle proteins in the cervix of HIV/HPV co-infected women. By using tissue micro-array (TMA) and immunohistochemistry to pRb, p27, VEGF and ELF-1, different CIN grades from HIV/HPV co-infected women were analyzed and compared to HPV/HIV-negative patients.

To our knowledge this is the first study to analyze the expression and protein alterations in different CIN grades in HIV and HPV co-infection correlating these protein alterations with histopathological and clinical features to evaluate putative prognostic marker potential.

## Methods

### Patients and tissue Samples

One hundred and sixty three fixed formalin, paraffin embedded samples with a histopathological diagnosis of CIN, cervicitis or cancer in cervical loop excisions and cone specimens were selected from the archives of the Department of Pathology of the Fernandes Figueira Institute (IFF), Fiocruz, Rio de Janeiro, Brazil. The IFF is a hospital that receives patients showing abnormal pap smears from Public Health Units of the State of Rio de Janeiro. Ninety-two cervical samples came from HPV positive women who were negative for HIV-1 and 37 were from HPV/HIV co-infected women. Thirty-four tissues diagnosed as nonspecific chronic cervicitis in women with no evidence of HPV or HIV-1 infection were used as controls.

Clinical data such as HIV-1 status, viral load, CD4/CD8 cell counts, age, other associated STDs and the use of anti-retroviral treatments (ARV/HAART) were obtained from patient files. The study was approved by the Ethical Committees Review Board of Evandro Chagas Clinical Research Institute and IFF, both from Fiocruz.

### Tissue Micro-Array (TMA) block construction

The TMA blocks were constructed as described by Pires et al, 2006 [13]. Briefly, all hematoxylin-eosin (HE) slides were re-examined by an experienced pathologist and two morphologic representative fields of higher CIN level were chosen and encircled with a marker pen. Two cores from each case were punched out from the donor blocks. Starting from the selected regions, the corresponding H&E slides were overlaid with a custom-built 16 gauge Becton-Dickinson PrecisionGlide^® ^hypodermic needle (1,1 mm^2 ^area). Afterwards cores were attached by double-side adhesive tape on a computer-generated paper grid affording alignment on the block mould, which was then filled with liquid paraffin. Three μm thick sections were obtained from an American Optical standard rotator microtome. Each block provided 40–50 slides, containing variable tissue core lengths. Only samples showing the original lesion were considered. The design of each block was detailed in a TMA map (spreadsheet), indicating the position and identification of each core. Normal tissue (placenta) and position-specific blank cores were adopted for orientation during microscopy analysis. A total of three TMA blocks were constructed, one with HPV/HIV co-infected cervical biopsies, another with controls together with CIN I and the third with CIN II/II, Ca and tumor.

### HPV Testing

The presence of HPV DNA was determined through a previously published *in situ *hybridization assay [14,15]. In brief, the probe cocktails can detect either the low risk HPV types (HPVs 6,11,42,43,44) or the high risk HPV types (HPVs 16,18,31,33,35,45,51,52,56,58,68) (Enzo Life Sciences, Farmingdale, NY, USA). The probe-target complex is detected due to the action of alkaline phosphatase on the chromogen nitroblue tetrazolium and bromochloroindolyl phosphate(NBT/BCIP) yielding a dark blue color with a pink counterstain for the HPV negative cells due to nuclear fast red.

### Immunohistochemistry (IHC)

IHC reactions were performed on TMA silane-coated slides (Sigma, St. Louis, MO, USA), dried overnight at 37°C and then dewaxed, rehydrated and treated in hydrogen peroxide 3% in methanol for 10 min to eliminate endogenous peroxidase activity. The LSAB system HRP (Dakocytomation, Carpinteria, CA, USA) method was adopted for immunolabelling. Tissue sections were sequentially incubated for 1 hour at room temperature with the specific antibodies (dilutions): anti-VEGF monoclonal antibody (1/400), anti-Elf-1 polyclonal antibody (1/300), anti-Rb monoclonal antibody (1/25) and anti-p27^Kp1 ^monoclonal antibody (1/20) (DakoCytomation, CA USA). A secondary biotinylated multilink antibody was incubated with the streptavidin-HRP conjugate for 30 min and the signal visualized with 3.3 diaminobenzidine (Sigma Chemical Co., St. Lois, MO, USA) and 85 μl of hydrogen peroxide 0.3%. Hematoxylin was the counterstain and the omission of the primary antibody, which eliminated the signal in each case, served as an additional control.

### Assessment of IHC slides and cell counting

The microscopic analysis of the slides was independently performed by two investigators. Positive stained cells were counted in the epithelium and stroma at 400× magnification. The total average area counted in the epithelium/stroma was: controls- 882/766 (SD 82,02) cells/mm^2^; CIN I – 867/642 (SD 159,09) cells/mm^2^; CIN II/III – 813/538 (SD 194,45) cells/mm^2^; carcinoma – 1.135/589 (SD 386,08) cells/mm^2^respectively. Digitalized photographs were taken with a Nikon COOLPIX Camera DP12, and the images stored in a computer-based software (ImagePro 4.5) for documentation.

### Statistical Analysis

Data analysis was carried out with the SAS System (SAS version 9.1, Cary, North Carolina, USA). Preliminary analyses verified that data did not follow a normal distribution, so we used non-parametric tests for comparisons. Mann-Whitney U, Kruskal Wallis and Dunn's tests were applied to compare means of positive cells in the epithelium and stroma which was then compared to peripheral CD4^+^T cells (< 200 and ≥ 200 cells/mm^3^), CIN grade, and presence/absence of HPV and/or HIV-1 infection. Differences were considered significant at p < 0.05.

## Results

### Histopathological data

HPV+/HIV-negative cervices (n = 92) were classified as low grade (CIN I; n = 33) or high grade/CIN (CIN II/III; n = 47). Twelve additional cases of invasive cancer, consisting of 3 cases of adenocarcinoma and 9 of squamous cell carcinoma (SCC) were included in the study. HPV+/HIV+ co-infected cervices (n = 37) were classified as low grade CIN (CIN I; n = 14) and high grade CIN (CIN II/III; n = 23).

### Immunohistochemical data: Overview

The expression of VEGF, Rb, Elf-1 and p27 in the epithelium of the cervical biopsies that were co-infected with HPV+/HIV+ was compared to the cervices infected by HPV alone. The results are summarized in Table 1 and Graphic 1 [see Additional file 1]. There was a statistically significant increase (p = 0.001) in the number of cells expressing VEGF and p27 in HIV+/HPV+ co-infection as compared to the HPV^+^/HIV^-^. Note that this increase was evident for both CIN I and CIN II/III. Table 2 provides a compilation of the comparisons of the number of epithelial cells expressing VEGF, p27, Rb, and Elf-1 in the cervicitis controls, CIN I, CIN II/III, and invasive carcinoma in women with HPV infection alone.

**Table 1:** Comparison between HPV and HPV/HIV co-infection with the different markers analyzed

	**NIC I**			**NICII/III**		
**Marker**	**HPV/HIV-**	**HPV/HIV+**	P-value*	**HPV/HIV-**	**HPV/HIV+**	P-value*
**VEGF-epit**						
Mean (SD)	74.9 (64.3)	161 (63.0)	0.001	89.6 (52.7)	169.5 (56.6)	< 0.001
Median (IQR)	70 (12 – 102)	161 (104 – 220)		82 (47 – 127)	162 (125–220)	
**(n)**	(32)	(12)		(41)	(22)	
**P27 epit**						
Mean (SD)	3.35 (20.5)	24.5 (34.4)	< 0.001	4.85 (15.2)	10.21 (12.0)	0.001
Median (IQR)	0 (0 – 0)	10 (5 – 33)		0 (0 – 3)	8 (0–16)	
**(n)**	(31)	(13)		(32)	(22)	
**Rb epit**						
Mean (SD)	11.5 (17.8)	33.6 (52.6)	0.141	3.41 (11.7)	11.5 (25.5)	0.110
Median (IQR)	0 (0 – 24)	19 (0 43)		0 (0 – 0)	0 (0–6)	
**(n)**	(29)	(13)		(41)	(21)	
**Elf-1 epit**						
Mean (SD)	48.6 (60.4)	93.1 (77.8)	0.191	133.5 (96.4)	98.5 (81.4)	0.509
Median (IQR)	20 (2 – 75)	69 (35–177)		99 (50 – 216)	69 (43–161)	
**(n)**	(27)	(14)		(35)	(21)	

*Mann-Whitney U test – The mean difference is significant at *p *< 0.05. IQR: Interquartile Range

**Table 2:** Protein expression in the epithelium (cells/mm^2^) of different studied markers in the uterine cervix from HPV+/HIV-negative infection

**Marker**	**VEGF**	**P27**	**Rb**	**Elf-1**
Chronic cervicitis				
Mean (SD)	36.73 (52.37)	0.19 (0.73)	12.5 (17.6)	76.26 (59.27)
Median (IQR)	0 (0 – 51.5)	0 (0–0)	0 (0 – 20)	65 (39 – 95)
(n)	n = 33	n = 32	n = 27	n = 27
CIN I				
Mean (SD)	102.5 (73.8)	8.25 (20.5)	17.9 (31.6)	63.8 (70.4)
Median (IQR)	74 (17.5 – 105.75)	0 (0 – 0)	0 (0 – 25.50)	20 (4 – 78)
	**			
(n)	n = 32	n = 31	n = 29	n = 27
CIN II/III				
Mean (SD)	127.7 (85.9)	9.26 (23.1)	4.8 (16.4)	115.7 (88.3)
Median (IQR)	77 (47 – 136)	0 (0 – 2.75)	0 (0 – 0)	99 (40 – 203)
	**		**	
(n)	n = 41	n = 32	= 41	n = 35
Tumor				
Mean (SD)	163.1 (187.36)	3.0 (3.0)	3.9 (12.3)	112 (87.6)
Median (IQR)	96 (43 – 248.5)	3 (0 – 6)	0 (0–.0)	101 (35. 5–166)
	**			
(n)	n = 9	n = 9	n = 10	n = 9
*p*-value*	< 0.001	0.047	0.003	0.005

Kruskal Wallis test – The mean difference is significant at *p *< 0.05. ** Dunn's test (α 0.05). IQR: Interquartile Range

### Elf-1 expression

**Figure 1:**
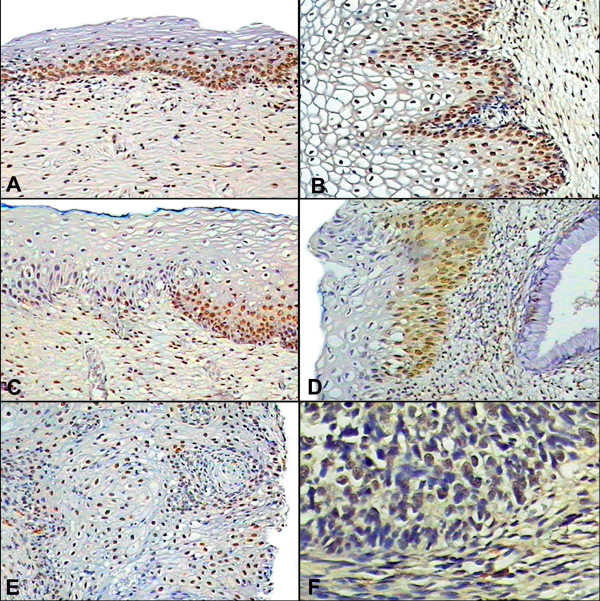
**Expression of Elf-1 in HIV+/HPV+ co-infected cervical tissues.** Panel A and B shows Elf-1 expression in ectocervical epithelium negative for CIN and CIN I respectively. Note that the signal is seen mainly in the basal layer of the epithelium (100× and 200×, respectively). Panel C – shows that the signal was evident only in the dysplastic cells in the CIN II lesion (100×). Panel D demonstrates that the Elf-1 signal tends to be more prominent in the squamous component as compared to the negative endocervical tissue (200×). Panel E – The signal in the squamous cell carcinoma tissues also localized to the neoplastic squamous cells (100×). However, neoplastic glandular cells demonstrated a multifocal signal as compared to the negative unremarkable endocervical tissue (panel F, 400×).

Elf-1 was evidenced in the cervical squamous epithelium, exhibiting a strong nuclear staining (Fig. 1A and 1B), particularly in the areas of CIN II/III. In non-neoplastic areas or without evidence of dysplasia, Elf-1 staining was scattered in the basal cell layer (Fig. 1C). In comparison, Elf-1 was not evident in the endocervix (Fig. 1D). The squamous cell cancer cases also showed a strong nuclear-based signal, whereas the adenocarcinoma cases displayed multifocal staining (Fig. 1E and 1F). HPV^+^/HIV^- ^infection showed statistical significant difference both in the epithelium (p = 0.005) and stroma (p = 0.009, data not shown). However, the Dunn's test failed to show differences between the controls and CIN I, CIN II/III and tumor. The epithelium of CIN II/III and tumor exhibited higher expression (115.7 and 112 cells/mm^2 ^respectively) compared to control and CIN I (76.26 and 63.8 cells/mm^2 ^respectively).

Although no significant differences appeared, the HPV+/HIV+ co-infected cervices, denoted a direct relation between increasing grade of the lesion and the intensity of staining in the epithelium as observed in CIN I (87.3 cells/mm^2^) and CIN II/III (104 cells/mm^2^). All invasive cancers in this study demonstrated high levels of proliferating cell nuclear staining indicative of cell proliferation.

### VEGF expression

**Figure 2:**
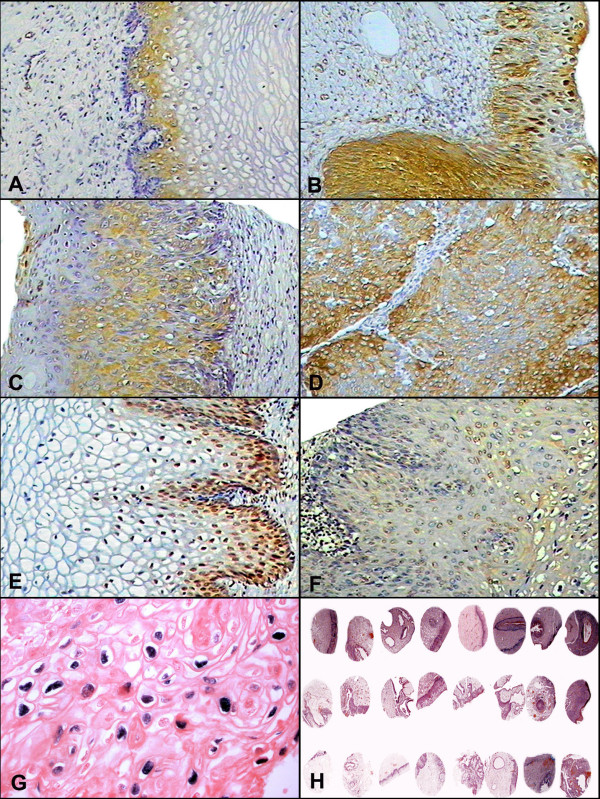
**Expression of VEGF, Rb, and p27 in cervical tissues.** Panel A shows VEGF expression in the negative control cervical tissue that was evident mostly in the basal layer of the epithelium (100×). VEGF expression was seen in the small vessels as well as in the cytoplasm of the dysplastic epithelium cells, particularly in the upper and intermediate layers in CIN I (panel B) and in CIN II/III (panel C),(100×). Panel D shows a squamous cell carcinoma showing a diffuse cytoplasmic positivity for VEGF in the neoplastic cells (100×). In panel E the strong nuclear expression of Rb is evident in CIN I, localizing mainly to the basal layer of the epithelium (200×). Panel F shows p27 nuclear staining in the superficial layer of the epithelium, mainly in the koilocytes of a CIN I lesion (200×). Panel G shows HPV DNA detection by in situ hybridization in the CIN II/III HPV TMA case (400×). Panel H shows a low magnification view of several Tissue Micro-Array cervical samples used in the present study (20×).

VEGF was observed in the endothelial cells of the small vessels as well as in the cytoplasm of the squamous cells, particularly in the upper and intermediate layers (Fig. 2A–2C). The intensity of the signal of the positive squamous cells was stronger in the CIN II/III lesions and carcinomas compared to the normal cervical tissue and CIN I (Fig. 2D). As already indicated, there was also a marked and significant increase in the number of positive cells with increasing CIN grade of the lesion in the HPV+/HIV-negative compared to controls, (p = < 0.001) by both Kruskal Wallis's and Dunn's test. HPV+/HIV+ co-infection cervices was markedly expressed in both CIN I and CIN II/III, however it not shown statistical significance.

### Rb expression

The Rb signal was evidenced by nuclear staining mainly in the basal layer of the epithelium (Fig. 2E). As evident from Table 1, the expression of Rb positive epithelial cells increased about 3-times in both CIN I and CIN II/III lesions when comparing the HPV+/HIV+ co-infected women to those infected by HPV alone. Interestingly, increasing grades of CIN were associated with decreased Rb epithelium expression in HPV+/HIV-negative cervices (average of cells/mm^2 ^in CIN I: 17.9, CIN II/III: 4.8, and tumor 3.9, p = 0.003) when compared to the cervicitis control (Table 2). This difference was significant using the Dunn's test in the category of CIN II/III compared to CIN I. Tumor cervices have shown a decreased expression, however no statistical difference was found probably caused by the reduced number of samples analyzed.

Separated analysis of HPV+/HIV+ co-infected cervices in the category of CIN II/III versus CIN I demonstrated significant difference (average of cells/mm^2 ^in CIN I: 28.9, CIN II/III: 10, p = 0.042), decreasing according to the CIN progression. Rare positive cells were seen in the stroma (p = 0.02, data not shown).

### p27 expression

p27 expression was evidenced as a nuclear signal in the superficial layer of the epithelium, mainly in the koilocytes (Fig. 2F). The stromal immunoreactive cells were inflammatory cells, with fewer numbers of positive cells evident in the stroma of squamous cell carcinomas. As discussed, p27 expression in epithelial cells was markedly increased in both CIN I and CIN II/III when comparing the HPV/HIV co-infected women to HPV infection alone (Table 1). When analyzing just HPV infected women compared to controls (Table 2), it was evident that p27 expression was markedly increased in both CIN I and CIN II/III (8.25 and 9.26 cells/mm^2^) and decreased in tumor (3.0 cells/mm^2^) cervices compared to the non-infected controls (0.19 cells/mm^2^).

### Clinical data of HIV/HPV co-infected women

#### Peripheral CD4 findings

Peripheral CD4 counts in HIV/HPV co-infected women did not demonstrate statistical differences (p = 0.81) when comparing low to high grade CIN lesions (Table 3). Table 3 summarizes the clinical data from HIV/HPV co-infected women. We then compared expression of VEGF, p27, Elf-1, and Rb relative to antiretroviral therapy (ARV). These comparisons showed one significant difference. Specifically, the Elf-1 transcriptor factor was less expressed in the epithelium group of patients not submitted to ARV treatment compared with those undergoing mono, dual and HAART (p < 0.005), (data not shown).

**Table 3:** Clinical and Histopathological data from HIV/HPV co-infected woman

**Histopathology**	**CIN I n = 14**	**CIN II/III/Ca n = 23**	**All N = 37**
**Log V. load**			
Mean (SD)	2.73 (1.42)	1.98 (1.35)	2.29 (1.40)
Median(IQR*)	1.90 (1.89–4.2)	1.84 (0.67–3.21)	1.89 (0.70–3.68)
Not available	5 (35.7%)	11 (47.8%)	15 (40.5%)
**CD4**			
Mean(SD)	336.3 (275.8)	269.1 (99.3)	300.7 (198.5)
Median(IQR*)	217.5 (107–571)	249 (220–344)	249 (150–383)
Not available	6 (42.8%)	14 (60.8%)	20 (54.0%)
**CD8**			
Mean(SD)	953.6 (338.8)	685.8 (214.5)	850.6 (317.3)
Median(IQR*)	980(743–1241)	619(509–896)	958(564–1061)
Not available	6(42.8%)	18(78.2)	24(64.8%)
**Age**			
Mean(SD)	37.3 (7.12)	35 (7.6)	36.0 (7.4)
Median(IQR*)	38(33–42)	35(30–44)	36.5(31–42.7)
Not available	1(7%)	0(0%)	1(2.7%)
**ARV**			
HAART	3(21.4%)	7(30.4%)	10(27%)
Mono/Dual	2(14.3%)	4 (17.4%)	6(16.2%)
No use	8(57.1%)	11(47.8%)	19(51.4%)
Not available	1(7.1%)	1(4.3%)	2(5.4%)

*IQR: Interquartile Range

#### HPV detection and TMA

Three different methods were employed to confirm the presence of HPV in the cervical samples. First, the hematoxylin and eosin stained slides were reviewed and all cervical biopsies with histologic findings of CIN were considered HPV positive given the large amount of data indicating that such lesions invariably contain the virus [12]. Second, HPV DNA was detected by the Hybrid capture HPV assay (Digene Diagnostic, USA) in the liquid based Pap smears in 12 of 37 HIV+/HPV+ co-infected women with CIN. Finally, HPV DNA *in situ *hybridization was conducted to document the presence of the virus in the TMA (Fig. 2G), and 67 were HPV DNA positive.

This comparative study underscored the value of the tissue Micro-Array (TMA) for possible putative prognostic markers, as it made possible the comparison of a large number of different CIN grade samples (Fig. 2H).

## Discussion

Our study confirmed the hypothesis that there is significantly altered expression of regulatory and cell cycle proteins in the cervix due to HIV-1 infection occurring in the context of a co-existing HPV infection. Specifically, the epithelial cells expressing VEGF and p27 was significantly increased with HIV+/HPV+ co infection compared to HPV infection alone for both CIN I and CIN II/III lesions. This effect by HIV-1 was additive to the HPV infection, as the latter per se was also associated with statistically increased expression of VEGF and p27 in CIN I-III and invasive cancers. Some explanations could be raised, including immunosuppression and the persistent HPV infection frequently seen in the HIV-1 infected patients. However, the mechanisms and the pathway by which HIV-1 can trigger these proteins require further molecular studies.

The modulation of p27, VEGF, and, perhaps, Rb expression may provide some insights into why HIV-1 infection of the cervix appears to be associated with an increased risk of cervical cancer in women co-infected by HPV. HIV-1 proteins can directly promote cancer growth by interfering with cellular functions. For example, the HIV *tat *protein has been shown to physically interact with the RB2/p130 tumor suppressor gene product [12] which may promote the effect of HPV oncoproteins. The effect of the HPV oncoproteins E6 and E7 may, in turn, be related to viral load [16]. On the other hand, the interactions between hr-HPV oncoproteins and molecules involved in cell cycle regulation are clearly related to the development of cervical cancer. Certainly other co-factors such as the HPV viral load may be predictive of the future risk of developing high-grade dysplasia or carcinoma, even in patients with negative Pap smears [16]. In HIV-infected women, HPV viral load is consistently high, which may explain in part the higher probability of high-grade dysplasia development [17]. In the present study we could not assay the local HPV viral load, but 57,5% of the HIV/HPV co-infected women exhibited high grade CIN. Persistent infection is the most important risk factor for initiating malignant transformation in the cervical epithelium, and a recent study reported that the timeframe from initial infection to pre-invasive and invasive disease appears to be at least 10–15 years [1].

Angiogenesis is important factor in the progression of CIN to invasive cancer and can be induced by many factors, including VEGF. In the present study we found that there was an increase in VEGF expression in the HPV only infected cervices, reaching the highest point in the invasive cancer cases, which is consistent with other report [4] that described a linear increasing from low grade CIN to high grade CIN and cancer lesions, suggesting that VEGF is a diagnostic marker for early stages of cervical carcinogenesis. However, in control cervices, we did find a low immunoreactivity both in the epithelium and stroma, perhaps explained by the fact that the vast majority of cervical tissues, even if taken from hysterectomy specimens where there is no suggestion of HPV infection, will exhibit inflammatory infiltrates in the cervix, that is, chronic cervicitis. In the HPV+/HIV+ co-infected cervices VEGF was highly expressed in both CIN I and CIN II/III, and showed no statistical difference.

The Elf-1 factor is a transcription factor that regulates a number of inducible lymphoid-specific genes and also in the development of tumor angiogenesis [5]. Elf-1 expression was statistically equivalent in the HPV alone and HPV/HIV-1 co-infection, suggesting that HIV-1 per se was not modulating its expression. Elf-1 expression did increase in CIN II/III and carcinoma lesions compared to the negative controls in HPV alone infected cervices and did reach statistical significance, suggesting that molecular events in the more progressed HPV infected cervices was increasing Elf-1 expression. Interestingly, Elf-1 was less expressed in patients without ARV treatment compared with those undergoing mono, dual or HAART (p < 0.005), possibly justified by the fact that some cellular transcription factors can bind to the HIV-1 5' long terminal repeat (LTR). However, the contribution of these factors to human immunodeficiency virus type 1 replication in infected individuals remains obscure [18].

Many tumor suppressor genes, most of them involved in the cell cycle, have been described, the functional loss of these suppressor gene pathways probably being the basis for cervical carcinogenesis. P27, an important cell cycle inhibitor protein, is involved in the growth and differentiation of the normal squamous epithelia of the uterine cervix, and controversial data have been described in the literature. Some authors have found that low levels of p27 are associated with poor prognosis and suggested that decreased p27 protein might contribute to the altered kinetics of the dysplastic and neoplastic cervical epithelium [19,20], whereas others have suggested that an increased and/or aberrant function of p27 expression may occur in invasive squamous cell carcinoma of the cervix [21]. Our data showed a marked increase in p27 infection by HPV alone in all CIN grades compared to controls, and decreased in tumor compared to any other group of CIN. One possibility to explain this increase is the blocking of p27 activity by the HPV oncogene E7 which binds p27 via its C terminus. However, the mechanisms by which the HIV would modify this activity is unknown. It is important to recall that cell cycle control is a multifactorial system and, for example, decreased Rb expression may increase cyclin E and cdk2 which, in turn, can overcome the tumor suppressive effect of increased p27 [22]. In this connection, our data show that there was a 3-fold increase in Rb expression with HIV-1 co-infection versus HPV infection alone, but this did not reach statistical significance, there may well be a stimulatory effect on Rb production due to HIV-1 co-infection in this context. This possibility will require additional studies. Nonetheless, since Rb expression decreased in CIN II/III and carcinoma in HPV only infected cervices compared to the negative controls, corroborating the previously published data [23,24]. Interestingly, HIV-1 co-infection appeared to rescue Rb expression which may, in turn, potentiate the increased expression of p27 [25]. However, this interaction by itself may not be sufficient for neoplastic transformation and other cofactors may be required. Nevertheless, it is likely that the deregulation of cellular genes and function by Tat can also cause abnormalities that may contribute to AIDS pathogenesis and to the development of AIDS-associated disorders [12].

This comparative study adopting Tissue Micro-Array for possible putative prognostic markers, made possible the comparison of a large number of different CIN grade samples, and demonstrated to be a useful research tool, primarily for immunohistochemistry procedures, in spite of some limitations. First, we could not assay high risk-HPV sub-typing in all samples, which prevented comparison between Hr-HPV types with the expression markers in the different CIN grades. Second, we cannot accurately determine the HPV type in these relatively small cores. Third, we decided to use nonspecific cervicitis as controls, as most transformation zone biopsies, even from women with no evidence of HPV or HIV-1 infection, show nonspecific cervicitis. Despite the limitations, we were able to document statistically significant changes due to HIV-1 co-infection in HPV infected cervices.

The neoplastic process of the uterine cervix occurs in the epithelium. In the present study we analyzed the expression of different markers both in the epithelium and stromal tissue, however we focused our analysis in the epithelium. Although VEGF demonstrated significant differences (p < 0.001) in low grade CIN compared to control in the HPV+/HIV-negative patients, this marker was also increased in high grade CIN, suggesting that it is not a good marker for CIN progression. Increasing grades of CIN were associated with decreased Rb expression cervices, however further studies with more infected cervices should be done in order to confirm these observations. Additionally, we suggest that many immune markers that have been studied as CIN progressors should also be evaluated in HIV/HPV co-infection, since this group of patients may have different clinical and immune outcomes.

## Conclusion

In conclusion, this study demonstrated a significant altered expression of regulatory and cell cycle proteins such as VEGF and p27 in cervical samples from HPV+/HIV+ co-infected compared to HPV+/HIV-negative women, suggesting that HIV-1 virus may be triggering these proteins which in turn could facilitate viral persistence and progression to an invasive tumor.

## Abbreviations

HPV: Human Papillomavirus; Rb: Retinoblastoma protein; VEGF: vascular endothelial growth factor; Elf: Eukariotic Translation Initiation Factors; TMA: Tissue Micro-Array; HIV: Human Immunodeficiency Syndrome; CIN: Cervical intra-epithelial neoplasia; ARV: antiretroviral; HAART: Highly active antiretroviral treatment; SCC: Squamous cell carcinoma; STD: Sexually Transmitted Disease.

## Competing interests

The authors declare that they have no competing interests.

## Authors' contributions

AFN conceived the study design, prepared the manuscript and was the main investigator. ARCP and SRS carried out the Tissue Micro-array studies and the immunohistochemistry analysis. GJN contributed to the immunohistochemical study and in the preparation of the manuscript. BG, AP and FR contributed toward the clinical patient selection and treatment. LSV participated in the statistical analysis, JRLS and CP participated in the discussion of the results and in the preparation of the manuscript. All authors have read and approved the final manuscript.

## Pre-publication history

The pre-publication history for this paper can be accessed here:



## Supplementary Material

Additional file 1 Click here for file

## References

[B1] Tjalma W, van Waes T, van den Eeden L, Bogers J (2005). Role of human papillomavirus in the carcinogenesis of squamous cell carcinoma and adenocarcinoma of the cervix. Best practice & Research Clin Obst Gynaecol.

[B2] Cho HN, Kim TY, Kim JW (2002). Alteration of cell cycle in cervical tumor associated with Human Papillomavirus – Cyclin-Dependent kinase inhibitors. Yonsei Med J.

[B3] Aggarwal BB, Shishodia S, Sandur SK, Pandey MK, Sethi G (2006). Inflammation and cancer: how hot is the link?. Biochem Pharmacol.

[B4] Branca M, Giorgi C, Santini D, Di Bonito L, Ciotti M, Benedetto A, Paba P, Costa S, Bonifacio D, Di Bonito P, Accardi L, Favalli C, Syrjanen K (2006). Aberrant expression of VEGF-C is related to grade of cervical intraepithelial neoplasia (CIN) and high risk HPV, but does not predict virus clearance after treatment of CIN or prognosis of cervical cancer. J Clin Pathol.

[B5] Huang X, Brown C, Ni W, Maynard E, Rigby AC, Oetthen P (2006). Critical role for the Ets transcription factor ELF-1 in the development of tumor angiogenesis. Blood.

[B6] Troncone G, Vetrani A, de Rosa G, Palombini L (1999). Cyclin dependent kinase inhibitor p27 expression in normal and neoplastic cervical epithelium. J Clin Pathol.

[B7] Benevolo M, Mottolese M, Marandino F, Vocaturo G, Sindico R, Piperno G, Mariani L, Sperduti I, Canalini P, Donnorso RP, Vocaturo A (2006). Immunohistochemical expression of p16(INK4a) is predictive of HR-HPV infection in cervical low-grade lesions. Mod Pathol.

[B8] Wentzensen N, von Knebel Doeberitz M (2007). Biomarkers in cervical cancer screening. Dis Markers.

[B9] Nicol AF, Fernandes AT, Grinsztejn B, Russomano F, E Silva JR, Tristao A, Perez M, Nuovo GJ, Martinez-Maza O, Bonecini-Almeida  M (2005). Distribution of immune cell subsets and cytokine-producing cells in the uterine cervix of human papillomavirus (HPV)-infected women: influence of HIV-1 coinfection. Diagn Mol Pathol.

[B10] Nicol AF, Nuovo GJ, Salomão-Estevez A, Grinsztein B, Tristão A, Russomano F, Lapa E Silva JR, Oliveira MP, Pirmez C (2008). Immune factors involved in the cervical immune response in the HIV/HPV co-infection. J Clin Pathol.

[B11] Gage JR, Sandhu AK, Nihira M, Bonecini-Almeida M da G, Cristoforoni P, Kishimoto T, Montz FJ, Martínez-Maza O (2000). Effects of human papillomavirus-associated cells on human immunodeficiency virus gene expression. Obstet Gynecol.

[B12] Nyagol J, Leucci E, Onnis A, De Falco G, Tigli C, Sanseverino F, Torriccelli M, Palummo N, Pacenti L, Santopietro R, Spina D, Gichangi P, Muchiri L, Lazzi S, Petraglia F, Leoncini L, Giordano A (2006). The effects of HIV-1 Tat protein on cell cycle during cervical carcinogenesis. Cancer Biol Ther.

[B13] Pires AR, da Matta Andreiuolo  F, de Souza SR (2006). TMA for all: a new method for the construction of tissue microarrays without recipient paraffin block using custom-built needles. Diagn Pathol.

[B14] Nicol AF, Nuovo GJ, Tor Savidge, Charalabos P, RT in situ PCR (2005). Protocols and Applications in Methods in Microbiology.

[B15] Nicol AF, Nuovo GJ, Wang Y, Grinsztejn B, Tristao A, Russomano F, Perez MA, Lapa e Silva JR, Fernandes AT, Gage JR, Martinez-Maza O, Bonecini-Almeida MG (2006). In situ detection of SOCS and cytokine expression in the uterine cervix from HIV/HPV coinfected women. Exp Mol Pathol.

[B16] Moberg M, Gustavsson I, Gyllensten U (2004). Type-specific associations of human papillomavirus load with risk of developing cervical carcinoma in situ. Int J Cancer.

[B17] Tjalma WA, Arbyan M, Paavonen J, van Waes TR, Bogers JJ (2004). Prophylactic human papillomavirus vaccines: the beginning of the end of cervical cancer. Int J Gynecol Cancer.

[B18] Estable MC, Bell B, Merzouki A, Montaner JS, O'Shaughnessy MV, Sadowski IJ (1996). Human immunodeficiency virus type 1 long terminal repeat variants from 42 patients representing all stages of infection display a wide range of sequence polymorphism and transcription activity. J Virol.

[B19] Goff BA, Sallin J, Garcia R, VanBlaricom A, Paley PJ, Muntz HG (2003). Evaluation of p27 in preinvasive and invasive malignancies of the cervix. Gynecol Oncol.

[B20] Shiozawa T, Shiohara S, Kanai M, Konishi I, Fujii S, Nikaido T (2001). Expression of the cell cycle regulator p27(Kip1) in normal squamous epithelium, cervical intraepithelial neoplasia, and invasive squamous cell carcinoma of the uterine cervix. Immunohistochemistry and functional aspects of p27(Kip1). Cancer.

[B21] Clarke B, Chetty R (2001). Cell cycle aberrations in the pathogenesis of squamous cell carcinoma of the uterine cervix. Gynecol Oncol.

[B22] Nam EJ, Kim JW, Kim SW, Kim YT, Kim JH, Yoon BS, Cho NH, Kim S (2007). The expressions of the Rb pathway in cervical intraepithelial neoplasia; predictive and prognostic significance. Gynecol Oncol.

[B23] Tringler B, Gup CJ, Singh M, Groshong S, Shroyer AL, Heinz DA, Shroyer KR (2004). Evaluation of p16INK4A and pRb expression in cervical squamous and glandular neoplasia. Hum Pathol.

[B24] Stiegler P, Kasten M, Giordano A (1998). The RB family of cell cycle regulatory factors. J Cell Biochem Suppl.

[B25] De Falco G, Bellan C, Lazzi S, Claudio P, La Sala D, Cinti C, Tosi P, Giordano A, Leoncini L (2003). Interaction between HIV-1 Tat and pRb2/p130: a possible mechanism in the pathogenesis of AIDS-related neoplasms. Oncogene.

