# Supplementary material for: Cell-cycle and suppressor proteins expression in uterine cervix in HIV/HPV co-infection: comparative study by tissue micro-array (TMA)
Source: BMC Cancer. 2008 Oct 7;8:289. doi: 10.1186/1471-2407-8-289 (PMC2577688; doi:10.1186/1471-2407-8-289)
Supplement: Additional file 1 [file 1471-2407-8-289-S1.doc]

**Graphic 1- Comparison between HPV/HIV co-infection and HPV with the different markers in the epithelium**
